# Estimated Pulse Wave Velocity as a Marker of Blood-Pressure-Dependent Arterial Load and Ventricular–Vascular Interaction in Severe Aortic Stenosis Before and After Transcatheter Aortic Valve Replacement

**DOI:** 10.3390/jcdd13040149

**Published:** 2026-03-25

**Authors:** Simina Mariana Moroz, Alina Gabriela Negru, Mirela Baba, Silvia Luca, Mihaela Valcovici, Alina Maria Lupu, Darius Buriman, Daniel-Dumitru Nișulescu, Ana Lascu, Daniel Florin Lighezan, Ioana Mozos

**Affiliations:** 1 Center for Advanced Research in Cardiovascular Pathology and Hemostaseology, “Victor Babeş” University of Medicine and Pharmacy, 300041 Timișoara, Romania; simina.moroz@umft.ro (S.M.M.);; 2 Doctoral School Medicine-Pharmacy, “Victor Babeş” University of Medicine and Pharmacy, 300041 Timișoara, Romania; mirela.baba@umft.ro (M.B.);; 3 Cardiology Department, “Victor Babeş” University of Medicine and Pharmacy, 300041 Timișoara, Romania; 4 Institute of Cardiovascular Diseases Timișoara, 300310 Timișoara, Romania; 5 Research Center of the Institute of Cardiovascular Diseases Timișoara, 300310 Timișoara, Romania; 6 Center for Translational Research and Systems Medicine, “Victor Babeş” University of Medicine and Pharmacy, 300041 Timișoara, Romania; 7 Discipline of Parasitology, Department of Infectious Diseases, Center for Diagnosis and Study of Parasitic Diseases, “Victor Babes” University of Medicine and Pharmacy, 300041 Timisoara, Romania; 8 Clinical Laboratory, Institute of Cardiovascular Diseases Timișoara, 300310 Timișoara, Romania; 9 Department of Functional Sciences-Pathophysiology, “Victor Babeş” University of Medicine and Pharmacy, 300041 Timișoara, Romania; 10 Department of Histology, Faculty of Medicine, “Vasile Goldiș” Western University of Arad, 310025 Arad, Romania; 11 Department of Internal Medicine I-Medical Semiotics I, “Victor Babeş” University of Medicine and Pharmacy, 300041 Timișoara, Romania

**Keywords:** TAVR, arterial stiffness, ePWV, aortic stenosis, ventricular–vascular interactions, arterial load, hemodynamic unloading

## Abstract

Background: Severe aortic stenosis (AS) increases left ventricular afterload and disrupts ventricular–vascular coupling. Transcatheter aortic valve replacement (TAVR) promptly relieves valvular obstruction, but its immediate effects on blood pressure-dependent arterial load and ventricular–vascular interactions are not fully clarified. Estimated pulse wave velocity (ePWV), derived from age and mean arterial pressure, is a convenient surrogate of global arterial load. The study aimed to assess ePWV before and after TAVR and its relationship with ventricular function and inflammatory biomarkers. Methods: In this retrospective observational study, 100 elderly patients with severe AS undergoing TAVR underwent detailed clinical, laboratory, and echocardiographic assessments before and after the procedure. Arterial stiffness was quantified using ePWV, while left ventricular geometry and systolic function were evaluated by standard echocardiography. Post-procedural reassessment was performed at hospital discharge (median 8 days after TAVR). Results: TAVR led to a modest but significant reduction in ePWV (from 12.79 ± 1.54 to 12.39 ± 1.54 m/s, *p* < 0.01) and improvement in left ventricular ejection fraction (LVEF) (from 44.89 ± 9.2% to 46.7 ± 7.95%, *p* < 0.01). Higher baseline ePWV correlated with unfavorable left ventricular remodeling and systolic dysfunction, and post-procedural ePWV remained linked to right ventricular performance. Before TAVR, ePWV and LVEF were both associated with inflammatory biomarkers, relationships that disappeared after intervention. Conclusions: Overall, ePWV functioned as an integrated measure of ventricular–vascular interaction and global hemodynamic load, though its interpretation post-TAVR requires caution due to direct blood pressure dependence and confounding by acute procedural inflammation.

## 1. Introduction

Aortic stenosis (AS) is the most prevalent valvular heart disease in the elderly population and represents a major cause of morbidity and mortality worldwide [1]. Beyond the mechanical obstruction of left ventricular outflow, AS is increasingly recognized as a complex cardiovascular condition involving profound alterations in arterial properties, ventricular structure, and ventricular–vascular interactions [2,3]. Chronic pressure overload leads not only to left ventricular hypertrophy and remodeling but also to changes in arterial stiffness, which further amplify afterload and impair cardiac performance [4,5,6,7].

Arterial stiffness plays a pivotal role in cardiovascular pathophysiology and is closely associated with aging, hypertension, and adverse cardiovascular outcomes [5,6,8]. In patients with AS, the coexistence of valvular obstruction and increased arterial stiffness creates a “double load” on the myocardium, contributing to ventricular remodeling and functional impairment [2,9]. However, the contribution of arterial stiffness to cardiac dysfunction in AS has historically been underappreciated.

TAVR has become an established therapeutic option for patients with severe AS across the entire surgical risk spectrum, improving survival and functional status [10,11,12]. While it induces rapid hemodynamic changes, its effects on arterial stiffness and ventricular–vascular interactions remain incompletely understood, with conflicting findings reported in the literature [13,14,15]. These discrepancies may reflect differences in patient characteristics, assessment methods, timing of measurements, and the complex interaction between vascular and myocardial remodeling.

Pulse wave velocity (PWV) is considered the gold standard, noninvasive method to assess arterial stiffness and is an independent predictor of cardiovascular events [4,6,16,17]. Pulse wave velocity, including brachial-ankle PWV, has strong prognostic value for cardiovascular events [17]. ePWV derived from age and mean arterial pressure, has emerged as a practical surrogate that allows arterial stiffness assessment without dedicated vascular equipment [16,17,18]. ePWV has demonstrated strong prognostic value in various cardiovascular settings and may be particularly useful in elderly and frail populations undergoing structural heart interventions. ePWV provides a practical surrogate for arterial stiffness assessment and correlates with directly measured carotid-femoral PWV, offering advantages in a routine clinical setting [18]. Nevertheless, data regarding the behavior of ePWV after TAVR and its relationship with ventricular remodeling and systemic biomarkers are scarce.

Beyond mechanical unloading, TAVR induces systemic physiological changes that may influence arterial load, including modifications in blood pressure profiles, neurohumoral activation, and inflammatory status [19]. Inflammatory markers, such as the neutrophil-to-lymphocyte ratio, have been increasingly recognized as indicators of cardiovascular risk and adverse remodeling [20,21]. The interaction between arterial stiffness, ventricular function, and systemic inflammation may therefore play an important role in cardiovascular adaptation following TAVR. However, despite the well-established hemodynamic benefits of the procedure, its impact on arterial stiffness and ventricular–vascular interactions remains incompletely understood, with conflicting findings reported in the literature.

In addition, TAVR is associated with an acute systemic inflammatory response related to vascular manipulation, prosthesis deployment, and peri-procedural stress. This transient inflammatory activation may influence vascular tone and myocardial performance in the early post-procedural period. Accordingly, in the present study, inflammatory markers were considered as contextual modulators of early hemodynamic and ventricular–vascular changes, rather than primary drivers of structural cardiovascular remodeling, in line with the study focus on early blood-pressure-dependent arterial load and ventricular–vascular functional changes.

Importantly, unlike prior studies focusing on directly measured pulse wave velocity or long-term vascular remodeling after TAVR, the present investigation specifically addresses early changes in blood pressure-dependent arterial load, as reflected by ePWV, and their relationship with ventricular structure, function, and systemic inflammatory response. By integrating echocardiographic, hemodynamic, and inflammatory parameters, this study seeks to clarify whether early reductions in ePWV after TAVR primarily reflect hemodynamic unloading rather than intrinsic arterial structural changes, and if ePWV captures ventricular–vascular interactions in the immediate post-procedural period.

**Figure 1:**
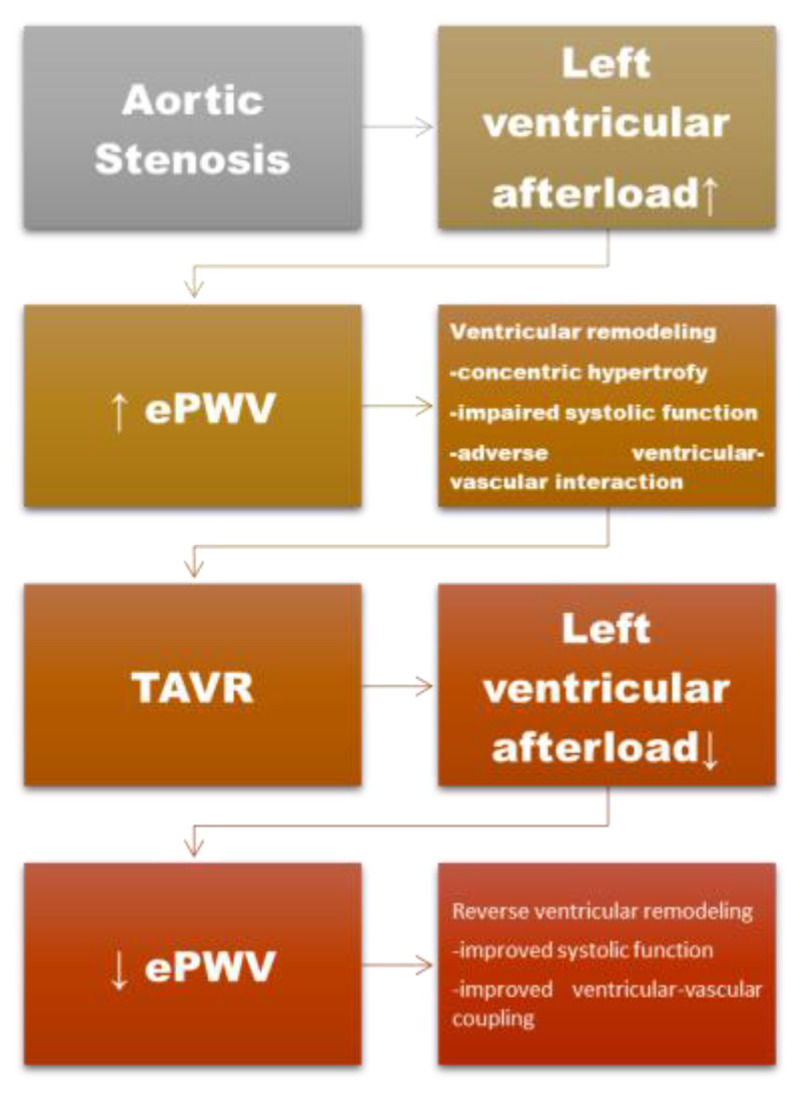
Conceptual figure of early ventricular–vascular remodeling in aortic stenosis and TAVR. In aortic stenosis, increased left ventricular afterload is associated with elevated arterial stiffness, reflected by increased ePWV, associated with adverse ventricular remodeling. Legend: TAVR—Transcatheter aortic valve replacement; ePWV—estimated pulse wave velocity, afterload ↑-increase in afterload; afterload ↓-decrease in afterload; ↓-decrease in estimated pulse wave velocity.

Therefore, the present study aimed to evaluate the impact of TAVR on arterial stiffness, as assessed by ePWV, and to explore its association with ventricular structure, ventricular function, and biological markers of inflammation. By focusing on early ventricular–vascular functional changes rather than isolated valvular hemodynamics, this study seeks to clarify the role of arterial stiffness in the pathophysiology of aortic stenosis and its evolution after TAVR. We hypothesized that relief of valvular obstruction by TAVR would be associated with early reductions in blood pressure-dependent arterial load and early ventricular–vascular functional changes (Figure 1).

Unlike previous studies focusing on long-term vascular remodeling, the present study specifically investigates early changes in blood pressure-dependent arterial load following TAVR, while acknowledging that such short-term observations primarily reflect acute hemodynamic adaptation.

## 2. Materials and Methods

### 2.1. Study Design

This retrospective observational study included consecutive elderly patients with severe aortic stenosis who underwent TAVR at a tertiary cardiovascular center. A total of 100 patients, who underwent TAVR between 2020 and 2023 in the Institute of Cardiovascular Diseases Timișoara, Romania, were enrolled based on the availability of complete clinical, biological, and echocardiographic data before and after the procedure. Severe aortic stenosis was diagnosed according to current guideline criteria [1,10]. Eligible patients had a confirmed diagnosis of severe AS, characterized by an aortic valve area (AVA) of ≤1.0 cm^2^, necessitating TAVR intervention (Table 1). All included patients were symptomatic and referred for TAVR according to current guideline recommendations. The study population reflects a real-world cohort of patients referred for TAVR. Low-flow, low-gradient aortic stenosis forms were not specifically excluded from the analysis. Baseline hemodynamic characterization included mean transvalvular gradient and LVEF. Patients with incomplete datasets or inadequate echocardiographic imaging were excluded from the analysis. The primary endpoint of the study was the change in ePWV after TAVR. Secondary endpoints included changes in ventricular function, ventricular geometry, and their association with inflammatory biomarkers.

The study protocol complied with the principles outlined in the Declaration of Helsinki and was approved by the local institutional ethics committee. Due to the retrospective nature of the study, the requirement for written informed consent was waived. The study was approved by the Ethics Committee for Scientific Research of the “Victor Babes” University of Medicine and Pharmacy (Nr. 59/18.12.2019 rev 202424). This retrospective observational study was conducted and reported in accordance with the STROBE (Strengthening the Reporting of Observational Studies in Epidemiology) guidelines.

### 2.2. Clinical Assessment

Baseline demographic data, cardiovascular risk factors, comorbidities, and clinical characteristics were collected from medical records. Blood pressure measurements were obtained using standard non-invasive sphygmomanometry in a resting condition. Systolic blood pressure, diastolic blood pressure, and mean arterial pressure were recorded before TAVR procedure and upon discharge from the hospital. Anti-hypertensive therapy was not systematically modified during hospitalization, minimizing its potential confounding effect on arterial stiffness assessment. Medication charts and discharge summaries were retrospectively reviewed to identify potential treatment adjustments.

Detailed patient demographics and clinical characteristics were presented in one of our previous studies [22].

### 2.3. Echocardiographic Evaluation

Echocardiographic assessments were conducted utilizing the Philips iE33 ultrasound system (Bothell, WA, USA). Echocardiographic evaluations were performed using standardized protocols. Patients were positioned in a left lateral decubitus posture to optimize acoustic window access. Standard 2D, M-mode, Doppler, and color flow imaging techniques were used. Transthoracic echocardiography was performed in all patients before TAVR and repeated after the procedure using standardized imaging protocols. Left ventricular systolic function was assessed by LVEF using conventional methods. LVEF was calculated using the Simpson’s biplane method in accordance with current echocardiographic guidelines. Left ventricular geometry was evaluated by measuring left ventricular end-diastolic diameter (LVEDD) and relative wall thickness. Standard transthoracic echocardiography included measurements of LVEF, left ventricular end-diastolic diameter (LVEDD), interventricular septal thickness (IVS), posterior wall thickness (PW), left atrial diameter (LA), tricuspid annular plane systolic excursion (TAPSE), pulmonary artery systolic pressure (PASP), and the TAPSE/PASP ratio. Additional structural and functional cardiac parameters were obtained according to current echocardiographic guidelines. All echocardiographic examinations were performed by experienced operators, according to standardized protocols. Procedural assessments were performed at hospital discharge, 8 days after TAVR.

### 2.4. Assessment of Arterial Stiffness

Arterial stiffness was assessed using ePWV calculated according to patient age and mean arterial pressure (MAP), using a validated equation [16]. This approach provides a reliable surrogate of carotid-femoral pulse wave velocity and has been shown to correlate with cardiovascular risk and vascular aging. ePWV was calculated both before and after TAVR to assess changes in arterial stiffness associated with the procedure. The ePWV as a marker of arterial stiffness was calculated using the equation: ePWV = 9.58748315543126 − 0.402467539733184 × age + 4.56020798207263 × 10^−3^ × age^2^ − 2.6207705511664 × 10^−5^ × age^2^ × MBP + 3.1762450559276 × 10^−3^ × age × MBP − 1.83215068503821 × 10^−2^ × MBP. MBP was calculated as DBP + 0.4 × (SBP − DBP), where DBP is the diastolic blood pressure and SBP is the systolic blood pressure. Because ePWV is mathematically derived from age and mean arterial pressure, it was interpreted in this study primarily as a composite marker of blood pressure-dependent arterial load rather than a direct measure of intrinsic arterial wall stiffness.

Accordingly, changes in ePWV after TAVR were analyzed as reflecting acute hemodynamic unloading and ventricular–arterial interaction, rather than structural vascular remodeling.

### 2.5. Biological and Inflammatory Markers

Venous blood samples were collected before TAVR and after the procedure as part of routine clinical evaluation. Hematological and biochemical parameters included hemoglobin, total white blood cell count, neutrophil count, lymphocyte count, liver enzymes, and creatinine. The neutrophil-to-lymphocyte ratio was calculated and selected as a readily available, routinely measured index of systemic inflammatory activation. Blood samples were obtained at baseline prior to the TAVR procedure and repeated at hospital discharge.

### 2.6. Statistical Analysis

Continuous variables are presented as mean ± standard deviation. Categorical variables are expressed as absolute numbers and percentages. Normality of data distribution was assessed before the analysis. Pre- and post-interventional data were compared using paired *t*-tests or non-parametric equivalents where appropriate. When data distribution did not meet normality assumptions, non-parametric comparisons were performed using the Wilcoxon signed-rank test. Correlations between ePWV and echocardiographic or biological parameters were assessed using Pearson correlation coefficients. Multivariable linear regression analyses were conducted to identify independent determinants of arterial stiffness before and after TAVR, adjusting for relevant clinical and echocardiographic variables. Variables included in multivariable regression models were selected a priori based on clinical relevance and prior evidence, including age, blood pressure parameters, and key echocardiographic measures of ventricular structure and function. To minimize the risk of overfitting given the sample size, the number of covariates included in each model was limited, and collinearity was carefully considered. Receiver operating characteristic (ROC) curve analysis was performed exploratorily to assess whether baseline ePWV could discriminate patients who developed early post-TAVR arrhythmia, as a surrogate of acute ventricular electrical instability. Because ePWV is mathematically derived from mean arterial pressure, blood pressure variables were not simultaneously included as independent predictors in regression models where ePWV served as the dependent variable in order to minimize potential multicollinearity.

Statistical significance was defined as a two-sided *p*-value < 0.05. All statistical analyses were performed using MedCalc® Statistical Software version 23.4.8 (MedCalc Software Ltd, Ostend, Belgium; https://www.medcalc.org; accessed on 3 July 2025). Given the exploratory nature of the study and the hypothesis-generating aim of evaluating ventricular–vascular interactions, no formal correction for multiple comparisons was applied. Correlation analyses were interpreted with emphasis on consistency of direction and physiological plausibility rather than isolated statistical significance.

## 3. Results

### 3.1. Baseline Characteristics

The study population consisted of 100 elderly patients with severe aortic stenosis, aged 78 ± 5.83 years. Most patients presented with multiple cardiovascular comorbidities, reflecting a high-risk, real-world TAVR population [22]. The study population had a balanced sex distribution (52% male, 48% female) and a high burden of cardiovascular comorbidities. The majority of patients were in New York Heart Association (NYHA) functional class II (66%) or III (29%), with a smaller proportion in class IV (4%), reflecting symptomatic severe aortic stenosis. Hypertension was highly prevalent, with most patients as grade II (69%) or grade III (26%), while diabetes mellitus was present in 32% of cases. Coronary artery disease was observed in 26% of patients, and carotid artery disease in 12%, indicating a significant atherosclerotic burden. Chronic obstructive pulmonary disease was present in 10% of patients, and a history of stroke was noted in 12%. Procedurally, Medtronic valves were used in the majority of cases (74%). Post-procedural complications were 7%, although new-onset arrhythmias were observed in 38% of patients, and permanent pacemaker implantation was required in 9%. Minor complications such as hematoma occurred in 2% of cases, while a new stroke was rare (1%). Medication adjustments during hospitalization were reported in 18% of patients. Additional comorbid conditions included abdominal aortic aneurysm (4%) and neoplasms (7%). Baseline echocardiographic evaluation revealed impaired left ventricular systolic function and evidence of ventricular remodeling, consistent with chronic pressure overload (Table 2). TAVR resulted in a marked reduction in transvalvular aortic gradient. The aortic mean gradient (Pmed) significantly decreased from 49.94 ± 15.16 mmHg before the procedure to 10.29 ± 5.07 mmHg after TAVR (*p* < 0.0001). Arterial stiffness assessed by ePWV was increased at baseline, indicating advanced vascular aging in this cohort, as shown in Table 2. RWT (Relative wall thickness) and TAPSE/PASP ratio before TAVR were 0.59 ± 0.13, and 0.47 ± 0.17 mm/mmHg, respectively.

### 3.2. Changes in Arterial Load After TAVR

**Figure 2:**
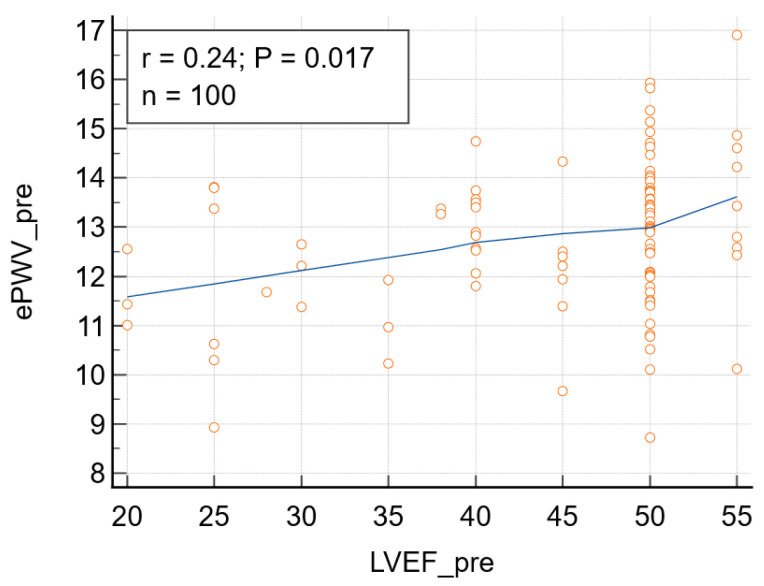
Before surgery: correlation between left ventricular ejection fraction (LVEF_pre) and estimated pulse wave velocity (ePWV): r = 0.24, *p* = 0.017, 95% confidence interval for r: 0.043 to 0.415. Blue line-format trendline; Yellow circles-individual patients’ data points.

Following TAVR, a significant reduction in ePWV was observed. ePWV decreased from 12.79 ± 1.54 m/s before the intervention to 12.39 ± 1.57 m/s after TAVR (*p* = 0.0001), demonstrating a consistent change in arterial load across the study population. The mean change in ePWV after TAVR was −0.40 m/s. Concomitantly, systolic blood pressure decreased by a mean of −7 mmHg, while LVEF increased by a mean of +1.8%. Although statistically significant, this modest reduction suggests an early hemodynamic unloading response following relief of valvular obstruction, rather than definitive evidence of structural vascular adaptation. At baseline, ePWV demonstrated significant correlations with left ventricular structural and functional parameters. Higher pre-interventional ePWV values were associated with larger left ventricular end-diastolic diameter and relative wall thickness, indicating a relationship between increased ePWV and adverse ventricular remodeling (Table 3). A significant association was also observed between baseline ePWV and left ventricular systolic function (Figure 2 and Table 3). Post-procedurally, the correlations with measures of ventricular structure and function lost statistical significance, but the correlation ePWV-RT was significant, suggesting that ePWV reflects persistent ventricular–vascular interactions even after correction of valvular obstruction (Table 3).

### 3.3. Ventricular Function and Remodeling

Right ventricular–pulmonary arterial coupling, assessed using the TAPSE/PASP ratio, showed preserved mean baseline values (0.47 ± 0.17). Left ventricular systolic function showed a modest but statistically significant improvement after TAVR. LVEF increased from 44.89 ± 9.20% pre-intervention to 46.70 ± 7.95% post-intervention (*p* = 0.0004). Structural parameters of left ventricular geometry also demonstrated favorable trends, supporting early ventricular functional changes following relief of valvular obstruction.

In multivariable linear regression analysis, pre-interventional ePWV was independently associated with left ventricular geometrical and functional parameters, even after adjustment for potential confounders (Table 4). These findings indicate that ePWV before TAVR is closely linked to ventricular remodeling rather than isolated hemodynamic burden. A significant reduction in the severity of associated valvular regurgitations was observed following TAVR. The proportion of patients with moderate-to-severe mitral, tricuspid, and aortic regurgitation decreased after the procedure. Mean mitral regurgitation grade declined from 2.22 ± 0.67 to 1.78 ± 0.91 (*p* < 0.0001), tricuspid regurgitation from 2.23 ± 0.73 to 1.76 ± 1.03 (*p* < 0.0001), and aortic regurgitation from 1.57 ± 0.64 to 0.95 ± 0.72 (*p* < 0.0001) (Table 2). Post-interventional ePWV was independently associated with right ventricular functional parameters, supporting the concept that arterial stiffness remains an integrated component of cardiovascular remodeling after TAVR. Significant correlations were found between ePWV before TAVR (ePWV_pre) and left ventricular end-diastolic diameter, LVEF, TAPSE/PASP ratio, and relative wall thickness (Table 3). ePWV calculated after TAVR (ePWV_post) was significantly correlated with RT, and borderline associated with RA, and LVEF assessed after TAVR.

In multiple regression analysis, left ventricular end-diastolic diameter (LVEDD) before TAVR remained a significant determinant of ePWV_pre (multiple R = 0.274, *p* = 0.0071) after adjusting for LVEF, TAPSE/PASP, Pmed, RT, RM and RA, respectively. TAPSE and RT_post were revealed as independent determinants of ePWV_post (multiple R = 0.372, *p* = 0.0008) (Table 4).

### 3.4. Biological and Inflammatory Parameters

Post-interventional biological assessment revealed a significant decrease in hemoglobin levels, consistent with procedural and periprocedural factors or hemodilution. Before TAVR, LVEF was inversely correlated with alanine aminotransferase (r = −0.329, *p* = 0.0008), aspartate aminotransferase (r = −0.282, *p* = 0.0045), hemoglobin levels (r = −0.225, *p* = 0.024), white blood cell count (r = −0.205, *p* = 0.0416), neutrophils (r = −0.207, *p* = 0.0399), monocytes (r = −0.201, *p* = 0.049), and NLR (r = −0.2145, *p* = 0.032). Markers of systemic inflammation showed dynamic changes after TAVR. The neutrophil-to-lymphocyte ratio increased post-intervention, reflecting an acute inflammatory response; however, its associations with ventricular function did not remain clinically relevant. Baseline ePWV showed modest but significant positive correlations with inflammatory markers, particularly white blood cell count, monocytes, and neutrophils, indicating an association between vascular properties, myocardial performance, and systemic inflammatory activation. These associations with inflammatory biomarkers lost significance after the intervention.

### 3.5. ePWV and Arrhythmia

Preoperative ePWV demonstrated no predictive value for postoperative arrhythmias, atrial fibrillation, and atrioventricular blocks, with area under the curve (AUC) values of 0.542, 0.524, and 0.526, respectively. This suggests that ePWV reflects hemodynamic load rather than electrical vulnerability.

## 4. Discussion

The present study provides several important findings, related to early changes in blood pressure-dependent arterial load in patients with aortic stenosis after TAVR. First, ePWV decreased modestly after TAVR, reflecting early changes in blood pressure-dependent arterial load rather than structural vascular remodeling. Second, baseline ePWV was associated with left ventricular geometry and systolic function, supporting its relationship with ventricular performance in severe aortic stenosis. Because post-procedural assessment was performed at hospital discharge, the observed changes should be interpreted as reflecting early hemodynamic adaptation rather than true structural ventricular or vascular remodeling.

A key finding of the present study is the observed shift in the determinants of ePWV from left ventricular-related parameters before TAVR to right ventricular functional indices after the intervention. This transition likely reflects the hemodynamic changes induced by relief of valvular obstruction, with a subsequent redistribution of ventricular loading conditions and an increased contribution of right ventricular-pulmonary arterial coupling in the early post-procedural phase.

The present study shows that TAVR is associated with early reduction in blood pressure-dependent load, as reflected by ePWV, alongside favorable changes in ventricular structure and function. Acute invasive hemodynamic studies have shown immediate reductions in left ventricular afterload following TAVR [19]. To our knowledge, this is one of the few studies specifically addressing early changes in ePWV and their relationship with early ventricular functional and geometric changes after TAVR. These findings support the concept that aortic stenosis is not solely a valvular disease but rather a complex cardiovascular condition involving dynamic interactions between the heart and the arterial system [2,3,9]. By focusing on arterial loads associated with ventricular -vascular coupling rather than isolated valvular hemodynamics, this study provides additional insight into the mechanism of cardiovascular recovery after TAVR. Previous studies evaluating arterial stiffness after TAVR have yielded heterogeneous results, with some reporting reductions in pulse wave velocity and others no or significant change, likely reflecting differences in methodology, timing of assessment, and patient populations [13,14,15].

Finally, the observed associations between ePWV, ventricular function, and inflammatory markers suggest that early post-TAVR adaptation involves a complex interplay between hemodynamic unloading and transient inflammatory activation.

### 4.1. Arterial Stiffness and TAVR

One of the key findings of this study is the significant decrease in ePWV, reflecting changes in blood pressure-dependent arterial load following TAVR. Although statistically significant, the magnitude of the reduction in ePWV was modest. The relatively small effect size likely reflects the mathematical dependence of ePWV on mean arterial pressure and should therefore be interpreted primarily as evidence of early hemodynamic unloading rather than clinically meaningful structural arterial remodeling. ePWV represents an integrated marker of vascular aging and blood pressure-dependent arterial load, which may be particularly relevant in elderly patients undergoing TAVR [16,17]. Although arterial stiffness contributes to left ventricular afterload, the two concepts are not interchangeable, and ePWV reflects a composite measure of vascular aging and blood pressure-dependent arterial properties [5,6,7]. Unlike direct measurements requiring specialized equipment, ePWV can be easily obtained from routinely collected clinical parameters, allowing widespread applicability in real-world practice [16].

In the context of aortic stenosis, where vascular stiffness, hypertension, and myocardial remodeling frequently coexist, ePWV may reflect the cumulative arterial burden experienced by the left ventricle [2,9]. This result contributes to an ongoing debate in the literature, as previous studies have reported heterogeneous effects of valve replacement on arterial stiffness [13,14,15]. Although statistically significant, the observed reduction in ePWV was modest in magnitude and should be interpreted as reflecting early hemodynamic unloading rather than clinically relevant structural arterial remodeling. The modest decrease in ePWV observed in our study aligns with findings that suggest relief of valvular obstruction may lead to improved arterial compliance and reduced vascular load. Previous studies have reported heterogeneous effects of TAVR on arterial stiffness, likely reflecting differences in patient characteristics, baseline blood pressure, and timing of assessment [13,14,15]. In contrast to most previous studies focusing on intermediate or long-term vascular changes, the present analysis emphasizes early adaptation after TAVR. The timing of arterial stiffness assessment after TAVR may critically influence observed results. Early post-intervention measurements may capture acute hemodynamic and inflammatory changes, while later assessments reflect longer-term vascular remodeling [13,14,15]. Differences in assessment timing may partially explain the heterogeneous findings reported in previous studies. While direct measures of carotid-femoral PWV remain the gold standard, ePWV has been validated in large cohorts and demonstrates strong associations with cardiovascular morbidity and mortality [4,6,16,17]. In the present cohort, the reduction in ePWV likely reflects a reduction in global arterial load following relief of chronic left ventricular pressure overload. By restoring forward flow and improving ventricular emptying, TAVR may contribute to reduced neurohumoral activation and sympathetic tone, leading to favorable changes in arterial compliance [19]. Importantly, the use of ePWV allows integration of both age-related vascular stiffness and blood pressure-dependent arterial load, making it particularly relevant in elderly patients undergoing TAVR [16,17]. Consequently, changes in ePWV following TAVR should be interpreted primarily as reflecting early hemodynamic changes related to blood pressure and ventricular–arterial interaction rather than structural vascular remodeling. However, because ePWV is derived from blood pressure and age, it does not directly measure ventricular–vascular coupling.

In this context, ePWV should be interpreted as a marker of global hemodynamic load associated with ventricular performance rather than a direct measure of ventricular–vascular coupling.

### 4.2. Ventricular Remodeling and Ventricular–Vascular Interaction

The association between arterial load and ventricular structural and functional parameters observed in this study highlights the bidirectional relationship between the myocardium and the arterial system [23]. Higher pre-intervention ePWV values were associated with adverse left ventricular geometry and impaired systolic function, supporting the concept that increased arterial stiffness contributes to ventricular remodeling beyond the effect of valvular obstruction alone [2,3,9]. Interestingly, the inverse correlation between baseline ePWV and LVED diameter may appear counterintuitive. However, in elderly patients with AS, ventricular dilation may occur in the context of reduced contractile reserve and lower systemic blood pressure. Given that ePWV is directly dependent on mean arterial pressure, lower blood pressure in these patients may result in lower calculated ePWV values. Therefore, this inverse association likely reflects the complex interplay between ventricular remodeling, hemodynamic status, and blood pressure rather than a direct causal relationship between arterial load, ventricular size and contractile reserve in elderly patients with advanced aortic stenosis. Accordingly, this finding should be interpreted with caution. The term reverse remodeling is used to describe early favorable trends in ventricular structure and function [24]. The positive association between ePWV and LVEF may reflect compensatory hyper-contractility in the setting of chronic pressure overload, rather than preserved myocardial health. Although statistically significant, the correlation between baseline ePWV and LVEF was modest in magnitude and should therefore be interpreted cautiously. In addition to improvements in arterial load, TAVR was associated with a significant reduction in the severity of concomitant mitral, tricuspid, and aortic regurgitation, likely reflecting early hemodynamic unloading rather than structural valve remodeling. After TAVR, ePWV remained associated with right ventricular function, suggesting that arterial stiffness continues to influence cardiac performance even after mechanical relief of the valve. Furthermore, the absence of significant discriminative capacity of ePWV for early arrhythmias reinforces the concept that ePWV primarily reflects hemodynamic afterload conditions rather than intrinsic myocardial electrical vulnerability. These findings emphasize that correction of valvular stenosis does not immediately normalize ventricular–vascular interactions and that arterial properties may modulate the extent and trajectory of reverse remodeling [23,24]. The regression results reinforce that estimated PWV is not just a vascular marker but an integrated readout of ventricular–vascular interaction, with determinants moving from LV geometry/function pre-TAVR to RV function and right-sided dimensions post-TAVR. Estimated PWV (arterial stiffness) appears to influence right-sided size and function after TAVR mainly via persistent abnormal ventricular–arterial coupling, altered ventricular interaction, and pulmonary vascular load, even after valve obstruction is relieved [25]. Elevated estimated PWV after TAVR likely maintains higher LV and pulmonary pressures, alters ventricular–arterial coupling, and via ventricular interdependence and pulmonary vascular load, contributes to adverse RV function and right-sided remodeling rather than allowing full right-heart reverse remodeling. Evidence is indirect but mechanistically coherent across TAVR, heart-failure, and population studies. A number of uncertainties still surround RV-pulmonary artery coupling and its potential role among TAVR candidates [26].

### 4.3. Inflammation, Arterial Stiffness, and Cardiac Function

Systemic inflammation has emerged as an important contributor to cardiovascular remodeling and adverse outcomes [20,21]. Calcific aortic stenosis is increasingly recognized as an interplay between valvular calcification, myocardial remodeling, and atrial pathology, interconnected through inflammatory, metabolic, and fibrotic pathways [27]. In this study, inflammatory markers, particularly the neutrophil-to-lymphocyte ratio, were found to be associated with ventricular function. The findings suggest that inflammation may represent a common pathway linking vascular stiffness and myocardial dysfunction in patients with aortic stenosis. Inflammatory processes have been implicated in both vascular stiffening and myocardial remodeling, and systemic inflammatory markers have been linked with adverse outcomes in structural heart disease [20,21]. The acute inflammatory response observed after TAVR is expected, given the invasive nature of the procedure [19]. It is important to emphasize that the inflammatory response observed after TAVR in the present study most likely reflects acute procedural and peri-procedural stress rather than chronic inflammatory cardiovascular remodeling. The marked post-procedural increase in the NLR ratio is consistent with previous reports describing transient inflammatory activation following transcatheter valve implantation. The present data support a concept in which systemic inflammation, LV dysfunction, and vascular stiffness are interrelated before TAVR, but TAVR partly “decouples” these axes: inflammation evolves dynamically, LV function remodels, and the simple baseline correlations with leukocyte counts and NLR largely lose clinical significance after intervention. TAVR acutely unloads the LV and modifies inflammatory signaling, which partly decouples hemodynamic and inflammatory drivers, but unmasks persistent vascular stiffness and pre-existing myocardial damage that continues to shape long-term outcomes. These findings support the concept that TAVR may transiently decouple the hemodynamic and inflammatory axes, whereby acute hemodynamic unloading occurs simultaneously with a procedure-related inflammatory response. This dissociation may contribute to the dynamic and sometimes discordant changes observed in vascular load markers and inflammatory parameters during the early post-procedural period. Not all authors mention an acute procedural inflammatory response. A hemodynamic-driven anti-inflammatory effect was also mentioned by Abu Khadija et al. after Transcatheter Aortic Valve Implantation [28].

### 4.4. Clinical Implications

The results of this study have several clinically relevant implications. First, they support the inclusion of arterial load assessment in the comprehensive evaluation of patients with aortic stenosis undergoing TAVR. ePWV represents a simple, noninvasive, and widely applicable tool that can provide complementary information beyond standard echocardiographic parameters, especially related to RV dysfunction, which strongly influences outcomes [16,17]. From a clinical perspective, these findings suggest that early post-TAVR changes in ePWV should be interpreted primarily as markers of global hemodynamic load rather than direct indicators of vascular remodeling. In elderly patients undergoing TAVR, ePWV may provide complementary information to blood pressure measurements by integrating age-related vascular properties and ventricular–arterial interaction, particularly in the immediate post-procedural period. This reinforces the importance of careful blood pressure management and monitoring, and vascular health optimization after TAVR to support ventricular recovery.

Second, the persistence of ventricular–vascular interactions after TAVR suggests that optimal patient management should not end with successful valve implantation. Attention to blood pressure control, vascular health, and systemic inflammation may be essential to maximize cardiovascular recovery and long-term outcomes [8,12].

Finally, the lack of predictive value of pre-interventional ePWV for early rhythm disturbances indicates that ePWV reflects global cardiovascular remodeling rather than short-term electrical complications, further supporting its role as a marker of structural and functional adaptation. Future studies should explore the longitudinal evolution of arterial load after TAVR and its relationship with long-term ventricular remodeling and clinical outcomes [13,14,15]. Integrating arterial stiffness assessment with echocardiographic and biological markers may enable a more personalized approach to post-TAVR management, identifying patients who may benefit from targeted vascular or anti-inflammatory therapies. The strengths of this study include a well-characterized real-world TAVR cohort, integrated assessment of arterial load and ventricular remodeling, and the use of a practical marker of vascular properties applicable in routine clinical practice. This approach provides complementary insight into cardiovascular adaptation beyond conventional valvular hemodynamic assessment. From a clinical perspective, early identification of patients with persistently increased arterial load after TAVR may help refine post-interventional management strategies focused on vascular health.

### 4.5. Study Limitations

Several limitations should be acknowledged. The retrospective design limits causal inference, and the single-center nature of the study may restrict generalization. Arterial stiffness was assessed using estimated rather than directly measured pulse wave velocity, however, ePWV has been extensively validated and offers practical advantages in elderly populations [25]. Additionally, long-term follow-up data were not available, preventing assessment of the prognostic impact of post-TAVR changes in arterial stiffness. Post-procedural measurements were performed at hospital discharge (approximately 8 days after TAVR) and therefore primarily reflect early hemodynamic adaptation rather than structural ventricular or vascular remodeling. Because ePWV is partially dependent on blood pressure, changes in arterial stiffness may reflect both intrinsic vascular properties and hemodynamic modifications after TAVR. No formal correction for multiple comparisons was applied due to the exploratory nature of the analyses. Cautious interpretation of ePWV in the early post-TAVR setting is required, especially when used as a surrogate for arterial stiffness. While ePWV may serve as a practical marker of global hemodynamic load, its dependence on blood pressure limits its ability to capture structural vascular changes in the absence of longer-term follow-up.

Inflammatory status was assessed using non-specific biomarkers, and serial measurements or additional inflammatory mediators, such as C-reactive protein or interleukins, were not available, limiting mechanistic interpretation of inflammatory-vascular interactions. No additional inflammatory biomarkers were systematically available due to the study’s retrospective nature. Because of the retrospective design, minor undocumented changes in antihypertensive or other vasoactive medications cannot be completely excluded and may represent a potential confounding factor influencing blood pressure values and calculated ePWV measurements. In addition, potential changes in diuretic therapy during hospitalization cannot be completely excluded and may have influenced loading conditions and echocardiographic parameters, including the severity of valvular regurgitation. Stroke volume index was not systematically available due to the retrospective nature of the dataset.

Given the number of correlation and regression analyses performed, the risk of type I error cannot be excluded, and the findings should therefore be interpreted with caution and considered hypothesis-generating. However, the Bonferroni correction, a widely used, simple method to control Type I errors in multiple comparisons, has several limitations, including a high likelihood of Type II errors (missing true effects), over-correction when tests are dependent, and reduced statistical power as the number of tests increases.

Other composite inflammatory indices, such as the systemic immune-inflammation index, were not evaluated, as they provide information largely overlapping with the NLR ratio and may be influenced by peri-procedural platelet variability and anticoagulation, potentially confounding interpretation in the early post-TAVR setting.

## 5. Conclusions

In patients with severe aortic stenosis undergoing TAVR, early post-procedural changes in ePWV were observed, primarily reflecting a reduction in blood pressure-dependent arterial load rather than true structural arterial remodeling. The decrease in ePWV appears to be driven by acute hemodynamic changes following relief of valvular obstruction, highlighting the close interaction between ventricular afterload and systemic arterial pressure in the immediate post-TAVR period.

Furthermore, the associations identified between ePWV, inflammatory markers, and echocardiographic parameters suggest that early vascular and ventricular responses after TAVR occur in the context of procedural stress and transient inflammatory activation, rather than established inflammatory-driven cardiovascular remodeling.

In particular, the post-procedural increase in NLR likely reflects an acute inflammatory response rather than a marker of chronic ventricular–vascular remodeling.

ePWV is not just a vascular marker but an integrated marker of ventricular–vascular interaction, with determinants moving from LV geometry/function pre-TAVR to RV function and right-sided dimensions post-TAVR. ePWV should therefore be interpreted not merely as a vascular marker, but as an integrated indicator of blood pressure-dependent arterial load and ventricular–vascular interaction, particularly in the early post-TAVR settings.

Future studies incorporating longitudinal assessments, direct arterial stiffness measurements, and clinical outcome correlations are warranted to define better the role of ventricular–arterial interactions and inflammatory responses in cardiovascular adaptation following TAVR.

## Figures and Tables

**Table 1:** Inclusion and exclusion criteria.

Criteria	Details
**Inclusion Criteria**	
Age	≥65 years
Diagnosis	Severe aortic stenosis with an aortic valve area ≤ 1 cm^2^
Data Availability	Available pre- and postoperative echocardiographic and laboratory data
**Exclusion Criteria**	
Prior Cardiac Surgery	History of cardiac surgery
Valve Disease	Severe mitral or tricuspid diseases
Pulmonary Disease	Severe chronic obstructive pulmonary disease (COPD)
Kidney Disease	Advanced chronic kidney disease (eGFR < 30 mL/min/1.73 m^2^)

**Table 2:** Values obtained for ePWV, ventricular function, and key biological parameters before and after TAVR.

Variable	Pre-TAVR	Post-TAVR	*p* Value
ePWV (m/s)	12.79 ± 1.54	12.39 ± 1.57	0.0001
LVEF (%)	44.89 ± 9.20	46.70 ± 7.95	0.0004
MR	2.22 ± 0.67	1.78 ± 0.91	<0.0001
RT	2.23 ± 0.73	1.76 ± 1.03	<0.0001
RA	1.57 ± 0.64	0.95 ± 0.72	<0.0001
Pmed (mmHg)	49.94 ± 15.16	10.29 ± 5.07	<0.0001
PASP (mmHg)	44.77 ± 10.2	39.51 ± 9.55	<0.0001
LVEDD (cm)	4.77 ± 0.69	4.48 ± 0.69	<0.0001
SBP (mmHg)	132 ± 21	125 ± 16	0.0015
DBP (mmHg)	75 ± 12	69 ± 12	0.0002
MBP (mmHg)	98 ± 13	92 ± 12	0.0001
Hemoglobin (g/dL)	13.09 ± 1.61	11.23 ± 1.68	<0.0001
WBC (×10^3^/µL)	8.58 ± 1.46	8.69 ± 2.57	0.092
Neutrophils (×10^3^/µL)	7.66 ± 1.01	7.02 ± 2.57	0.1004
NLR (%)	3.57 ± 1.85	10.25 ± 7.12	<0.0001
Creatinine (mg/dL)	1.14 ± 0.62	1.10 ± 0.76	0.208
ALAT (U/L)	26.7 ± 14.16	27.63 ± 43.65	0.807
ASAT (U/L)	25.69 ± 12.89	37.19 ± 66.55	0.089

Abbreviations: ePWV—estimated pulse wave velocity; LVEF—left ventricular ejection fraction; LVEDD—left ventricular end-diastolic diameter; SBP—Systolic blood pressure; DBP—Diastolic blood pressure; MBP—Mean arterial blood pressure; NLR—Neutrophil-to-lymphocyte ratio, MR—mitral regurgitation; RT—tricuspid regurgitation; RA—aortic regurgitation; Pmed—aortic mean gradient; PASP—pulmonary artery systolic pressure; ALAT—alanine aminotransferase; ASAT—aspartate aminotransferase; WBC—white blood cell count.

**Table 3:** Correlations between ePWV and echocardiographic variables and inflammatory biomarkers, respectively, before and after TAVR.

Correlations Between	r (*p*)
ePWV_pre-PASP	−0.18 (0.068)
ePWV_pre-RWT	0.22 (0.025)
ePWV_pre-LVEF_pre	0.24 (0.017)
ePWV_pre-TAPSE/PASP	0.20 (0.042)
ePWV_pre-LVEDD	−0.28 (0.005)
ePWV_pre-WBC_pre	0.23 (0.0234)
ePWV_pre-Neutrophils_pre	0.24 (0.0162)
ePWV_pre-Monocytes_pre	0.24 (0.0189)
ePWV_pre-N/L	0.21 (0.032)
ePWV_post-RT_post	0.22 (0.026)
ePWV_post-RA_post	0.19 (0.058)
ePWV_post-LVEF_post	0.19 (0.055)

Abbreviations: ePWV_pre—estimated pulse wave velocity before TAVR; ePWV_post—estimated pulse wave velocity after TAVR, PASP—pulmonary artery systolic pressure; RWT—Relative wall thickness; LVEF_pre—left ventricular ejection fraction before TAVR; LVEF_post—left ventricular ejection fraction after TAVR; TAPSE/PASP—index of right ventricle–pulmonary artery coupling; RT_post—tricuspid regurgitation after TAVR; RA_post—aortic regurgitation after TAVR; WBC—white blood cell count, NLR—Neutrophil-to-lymphocyte ratio.

**Table 4:** Multiple regression analysis.

Dependent Variable	Independent Variables	Multiple R	R Square	Adjusted R Square	95% Confidence Interval	Significance
ePWV_pre	LVEDD Adjusted for TAPSE/PASP, LVEF_pre, RA_pre, RT_pre, RM_pre, Pmed_pre	0.2743	0.07524	0.06529	−1.0421 to −0.1683	0.0071
ePWV_post	TAPSE (*p* = 0.0038) RT_POST (*p* = 0.0014) Adjusted for LVEDD	0.3722	0.1386	0.1204	0.04190 to 0.2115 and 0.1990 to 0.8057	0.0008

Abbreviations: ePWV_pre—estimated pulse wave velocity before TAVR; LVEF_pre—left ventricular ejection fraction before TAVR; LVEDD—left ventricular end-diastolic diameter; PASP_pre—pulmonary artery systolic pressure before TAVR; RA_pre—aortic regurgitation before TAVR, RT_pre—tricuspid regurgitation before TAVR; RT_post—tricuspid regurgitation after TAVR; Pmed_pre—aortic mean gradient before TAVR; TAPSE—tricuspid annular plane systolic excursion; TAPSE/PASP—index of right ventricle–pulmonary artery coupling.

## Data Availability

The data presented in this study are available on request from the corresponding author.

## Abbreviations

The following abbreviations are used in this manuscript:

ALAT	Alanine aminotransferase
AS	Aortic stenosis
ASAT	Aspartate aminotransferase
AVR	Aortic valve replacement
BP	Blood pressure
cfPWV	Carotid-femoral pulse wave velocity
CI	Confidence interval
CV	Cardiovascular
DBP	Diastolic blood pressure
ePWV	Estimated pulse wave velocity
ESC	European Society of Cardiology
ESH	European Society of Hypertension
HTN	Hypertension
LVEF	Left ventricular ejection fraction
LV	Left ventricle/Left ventricular
LVEDD	Left ventricular end-diastolic diameter
MAP	Mean arterial pressure
MR	Mitral regurgitation
NLR	Neutrophil-to-lymphocyte ratio
PASP	Pulmonary artery systolic pressure
PH	Pulmonary hypertension
Pmed	Aortic mean gradient
PWV	Pulse wave velocity
RA	Aortic regurgitation
RT	Tricuspid regurgitation
RV	Right ventricle/Right ventricular
RWT	Relative wall thickness
SAVR	Surgical aortic valve replacement
SBP	Systolic blood pressure
TAPSE	Tricuspid annular plane systolic excursion
TAPSE/PASP	Index of right ventricle–pulmonary artery coupling
TAVR	Transcatheter aortic valve replacement
WBC	White blood cell count
